# Supplementary material for: Evaluation of the ELITe InGenius PCR assay compared with immunofluorescence for Pneumocystis jirovecii detection in respiratory samples
Source: Eur J Clin Microbiol Infect Dis. 2026 Mar 30;45(7):2085–96. doi: 10.1007/s10096-026-05474-9 (PMC13328313; doi:10.1007/s10096-026-05474-9)
Supplement: Supplementary file 1 [file 10096_2026_5474_MOESM1_ESM.docx]

# Supplemental material

Figure S1: PCR copy number distribution (log10 scale) stratified by IF assay supplier. a) Primary data b) Reexamination (RXM)

Figure S2: Flowchart showing data processing of discrepant results in microscopy and PCR analysis using the assay’s limit of detection (LoD; 97 cp/ml). The unadjusted primary data (orange) includes six IF+/PCR- and 41 IF-/PCR+ samples, 13 of which had high PCR positivity and thus were selected for further reexamination. 

IF reexamination (green) was performed in three IF+/PCR- and eight IF-/PCR+ cases with enough available material left and revealed three true-negative and two true-positive samples. The 'Clinical reevaluation' (blue) revealed further three confirmed PJP cases with negative IF. The final dataset, 'No false negatives' (violet), further removes the remaining three false-negative PCR cases. 

Abbreviations: PPV, positive predictive value; NPV, negative predictive value

Ein Bild, das Text, Diagramm, Reihe, Screenshot enthält.  KI-generierte Inhalte können fehlerhaft sein.Figure S3: ROC curves comparing PCR performance across four datasets: “Primary data”, “Reexamination” (RXM), Clinical reevaluation  (CRVL), and “No false negatives” (NFN).

Table S1: Overview of primary data including sample type, PCR and microscopy results, as well as additional sensitivity analyses. 

| NR | MAT | cp/ml | Ct value | microscopy | Gie | Tol | IF | IFA | RXM | CR | NFN |
| --- | --- | --- | --- | --- | --- | --- | --- | --- | --- | --- | --- |
| 1 | BAL | 5513098 | 20.46 | 1 | 1 | 1 | 1 | Bio-Rad | 1 | 1 | 1 |
| 2 | BAL | 486 | 34.57 | 0 | 0 | 0 | 0 | Bio-Rad | 0 | 0 | 0 |
| 3 | BAL | 0 | 46.00 | 0 | 0 | 0 | 0 | Bio-Rad | 0 | 0 | 0 |
| 4 | SP | 10000000 | 17.61 | 1 | 1 | 1 | 1 | Bio-Rad | 1 | 1 | 1 |
| 5 | SP | 0 | 46.00 | 0 | 0 | 0 | 0 | Bio-Rad | 0 | 0 | 0 |
| 6 | BAL | 34573 | 28.12 | 1 | 0 | 1 | 1 | Bio-Rad | 1 | 1 | 1 |
| 7 | SP | 28181 | 28.43 | 0 | 0 | 0 | 0 | Bio-Rad | 0 | 0 | 0 |
| 8 | SP | 526 | 34.45 | 0 | 0 | 0 | 0 | Bio-Rad | 0 | 0 | 0 |
| 9 | SP | 0 | 46.00 | 0 | 0 | 0 | 0 | Bio-Rad | 0 | 0 | 0 |
| 10 | BAL | 0 | 46.00 | 0 | 0 | 0 | 0 | Bio-Rad | 0 | 0 | 0 |
| 11 | SP | 0 | 40.20 | 0 | 0 | 0 | 0 | Bio-Rad | 0 | 0 | 0 |
| 12 | SP | 8866 | 30.18 | 0 | 0 | 0 | 0 | Bio-Rad | 0 | 0 | 0 |
| 13 | BAL | 0 | 46.00 | 0 | 0 | 0 | 0 | Bio-Rad | 0 | 0 | 0 |
| 14 | SP | 20163 | 28.94 | 0 | 0 | 0 | 0 | Bio-Rad | 0 | 0 | 0 |
| 15 | BAL | 0 | 46.00 | 0 | 0 | 0 | 0 | Bio-Rad | 0 | 0 | 0 |
| 16 | BAL | 373 | 34.97 | 0 | 0 | 0 | 0 | Bio-Rad | 0 | 0 | 0 |
| 17 | BAL | 0 | 38.34 | 0 | 0 | 0 | 0 | Bio-Rad | 0 | 0 | 0 |
| 18 | BAL | 0 | 46.00 | 0 | 0 | 0 | 0 | Bio-Rad | 0 | 0 | 0 |
| 19 | SP | 0 | 46.00 | 0 | 0 | 0 | 0 | Bio-Rad | 0 | 0 | 0 |
| 20 | SP | 0 | 38.57 | 0 | 0 | 0 | 0 | Bio-Rad | 0 | 0 | 0 |
| 21 | SP | 0 | 46.00 | 0 | 0 | 0 | 0 | Bio-Rad | 0 | 0 | 0 |
| 22 | BAL | 0 | 46.00 | 0 | 0 | 0 | 0 | Bio-Rad | 0 | 0 | 0 |
| 23 | BAL | 0 | 39.28 | 0 | 0 | 0 | 0 | Bio-Rad | 0 | 0 | 0 |
| 24 | BAL | 0 | 46.00 | 0 | 0 | 0 | 0 | Bio-Rad | 0 | 0 | 0 |
| 25 | BAL | 0 | 46.00 | 0 | 0 | 0 | 0 | Bio-Rad | 0 | 0 | 0 |
| 26 | BAL | 0 | 46.00 | 0 | 0 | 0 | 0 | Bio-Rad | 0 | 0 | 0 |
| 27 | SP | 0 | 46.00 | 0 | 0 | 0 | 0 | Bio-Rad | 0 | 0 | 0 |
| 28 | BAL | 0 | 46.00 | 0 | 0 | 0 | 0 | Bio-Rad | 0 | 0 | 0 |
| 29 | BAL | 0 | 46.00 | 0 | 0 | 0 | 0 | Bio-Rad | 0 | 0 | 0 |
| 30 | SP | 0 | 46.00 | 0 | 0 | 0 | 0 | Bio-Rad | 0 | 0 | 0 |
| 31 | BAL | 0 | 41.61 | 0 | 0 | 0 | 0 | Bio-Rad | 0 | 0 | 0 |
| 32 | SP | 0 | 46.00 | 0 | 0 | 0 | 0 | Bio-Rad | 0 | 0 | 0 |
| 33 | BAL | 4110 | 31.35 | 0 | 0 | 0 | 0 | Bio-Rad | 0 | 0 | 0 |
| 34 | SP | 36713 | 27.99 | 0 | 0 | 0 | 0 | Bio-Rad | 0 | 0 | 0 |
| 35 | BAL | 0 | 46.00 | 0 | 0 | 0 | 0 | Bio-Rad | 0 | 0 | 0 |
| 36 | BAL | 0 | 41.10 | 0 | 0 | 0 | 0 | Bio-Rad | 0 | 0 | 0 |
| 37 | BAL | 0 | 46.00 | 0 | 0 | 0 | 0 | Bio-Rad | 0 | 0 | 0 |
| 38 | SP | 0 | 46.00 | 0 | 0 | 0 | 0 | Bio-Rad | 0 | 0 | 0 |
| 39 | SP | 0 | 37.52 | 0 | 0 | 0 | 0 | Bio-Rad | 0 | 0 | 0 |
| 40 | SP | 0 | 46.00 | 0 | 0 | 0 | 0 | Bio-Rad | 0 | 0 | 0 |
| 41 | BAL | 0 | 46.00 | 0 | 0 | 0 | 0 | Bio-Rad | 0 | 0 | 0 |
| 42 | BAL | 0 | 46.00 | 0 | 0 | 0 | 0 | Bio-Rad | 0 | 0 | 0 |
| 43 | BAL | 93069 | 26.15 | 1 | 0 | 0 | 1 | Bio-Rad | 1 | 1 | 1 |
| 44 | SP | 0 | 46.00 | 0 | 0 | 0 | 0 | Bio-Rad | 0 | 0 | 0 |
| 45 | BAL | 223 | 35.62 | 0 | 0 | 0 | 0 | Bio-Rad | 0 | 0 | 0 |
| 46 | BAL | 0 | 46.00 | 0 | 0 | 0 | 0 | Bio-Rad | 0 | 0 | 0 |
| 47 | BAL | 0 | 46.00 | 0 | 0 | 0 | 0 | Bio-Rad | 0 | 0 | 0 |
| 48 | SP | 0 | 46.00 | 0 | 0 | 0 | 0 | Bio-Rad | 0 | 0 | 0 |
| 49 | SP | 225 | 35.60 | 0 | 0 | 0 | 0 | Bio-Rad | 0 | 0 | 0 |
| 50 | BAL | 0 | 46.00 | 1 | 0 | 0 | 1 | Bio-Rad | 0 | 0 | 0 |
| 51 | BAL | 0 | 46.00 | 0 | 0 | 0 | 0 | Bio-Rad | 0 | 0 | 0 |
| 52 | BAL | 0 | 46.00 | 0 | 0 | 0 | 0 | Bio-Rad | 0 | 0 | 0 |
| 53 | BAL | 0 | 46.00 | 0 | 0 | 0 | 0 | Bio-Rad | 0 | 0 | 0 |
| 54 | BAL | 0 | 46.00 | 0 | 0 | 0 | 0 | Bio-Rad | 0 | 0 | 0 |
| 55 | BAL | 0 | 46.00 | 0 | 0 | 0 | 0 | Bio-Rad | 0 | 0 | 0 |
| 56 | BAL | 522330 | 23.44 | 1 | 0 | 0 | 1 | Bio-Rad | 1 | 1 | 1 |
| 57 | BAL | 0 | 46.00 | 0 | 0 | 0 | 0 | Bio-Rad | 0 | 0 | 0 |
| 58 | SP | 0 | 37.09 | 0 | 0 | 0 | 0 | Bio-Rad | 0 | 0 | 0 |
| 59 | BAL | 0 | 39.13 | 0 | 0 | 0 | 0 | Bio-Rad | 0 | 0 | 0 |
| 60 | BAL | 0 | 46.00 | 0 | 0 | 0 | 0 | Bio-Rad | 0 | 0 | 0 |
| 61 | BAL | 0 | 46.00 | 0 | 0 | 0 | 0 | Bio-Rad | 0 | 0 | 0 |
| 62 | BAL | 0 | 46.00 | 0 | 0 | 0 | 0 | Bio-Rad | 0 | 0 | 0 |
| 63 | BAL | 0 | 39.02 | 0 | 0 | 0 | 0 | Bio-Rad | 0 | 0 | 0 |
| 64 | BAL | 0 | 46.00 | 0 | 0 | 0 | 0 | Bio-Rad | 0 | 0 | 0 |
| 65 | BAL | 0 | 46.00 | 0 | 0 | 0 | 0 | Bio-Rad | 0 | 0 | 0 |
| 66 | BAL | 0 | 46.00 | 0 | 0 | 0 | 0 | Bio-Rad | 0 | 0 | 0 |
| 67 | BAL | 0 | 46.00 | 0 | 0 | 0 | 0 | Bio-Rad | 0 | 0 | 0 |
| 68 | BAL | 0 | 46.00 | 0 | 0 | 0 | 0 | Bio-Rad | 0 | 0 | 0 |
| 69 | BAL | 0 | 46.00 | 0 | 0 | 0 | 0 | Bio-Rad | 0 | 0 | 0 |
| 70 | BAL | 0 | 46.00 | 0 | 0 | 0 | 0 | Bio-Rad | 0 | 0 | 0 |
| 71 | BAL | 0 | 46.00 | 0 | 0 | 0 | 0 | Bio-Rad | 0 | 0 | 0 |
| 72 | SP | 0 | 46.00 | 0 | 0 | 0 | 0 | Bio-Rad | 0 | 0 | 0 |
| 73 | BAL | 0 | 46.00 | 0 | 0 | 0 | 0 | Bio-Rad | 0 | 0 | 0 |
| 74 | BAL | 0 | 46.00 | 0 | 0 | 0 | 0 | Bio-Rad | 0 | 0 | 0 |
| 75 | SP | 17287749 | 17.95 | 1 | 0 | 0 | 1 | Bio-Rad | 1 | 1 | 1 |
| 76 | BAL | 225 | 35.61 | 0 | 0 | 0 | 0 | Bio-Rad | 0 | 0 | 0 |
| 77 | BAL | 1422 | 32.71 | 0 | 0 | 0 | 0 | Bio-Rad | 0 | 0 | 0 |
| 78 | SP | 14687 | 29.05 | 0 | 0 | 0 | 0 | Bio-Rad | 0 | 0 | 0 |
| 79 | SP | 0 | 46.00 | 0 | 0 | 0 | 0 | Bio-Rad | 0 | 0 | 0 |
| 80 | SP | 0 | 46.00 | 0 | 0 | 0 | 0 | Bio-Rad | 0 | 0 | 0 |
| 81 | SP | 400151 | 24.13 | 0 | 0 | 0 | 0 | Bio-Rad | 0 | 0 | 0 |
| 82 | BAL | 0 | 46.00 | 0 | 0 | 0 | 0 | Bio-Rad | 0 | 0 | 0 |
| 83 | BAL | 0 | 46.00 | 0 | 0 | 0 | 0 | Bio-Rad | 0 | 0 | 0 |
| 84 | SP | 0 | 46.00 | 0 | 0 | 0 | 0 | Bio-Rad | 0 | 0 | 0 |
| 85 | SP | 0 | 46.00 | 0 | 0 | 0 | 0 | Bio-Rad | 0 | 0 | 0 |
| 86 | SP | 0 | 37.14 | 0 | 0 | 0 | 0 | Bio-Rad | 0 | 0 | 0 |
| 87 | BAL | 0 | 46.00 | 0 | 0 | 0 | 0 | Bio-Rad | 0 | 0 | 0 |
| 88 | SP | 0 | 46.00 | 0 | 0 | 0 | 0 | Bio-Rad | 0 | 0 | 0 |
| 89 | SP | 0 | 46.00 | 0 | 0 | 0 | 0 | Bio-Rad | 0 | 0 | 0 |
| 90 | BAL | 0 | 46.00 | 0 | 0 | 0 | 0 | Bio-Rad | 0 | 0 | 0 |
| 91 | BAL | 0 | 46.00 | 0 | 0 | 0 | 0 | Bio-Rad | 0 | 0 | 0 |
| 92 | BAL | 79433 | 26.61 | 1 | 0 | 1 | 1 | Bio-Rad | 1 | 1 | 1 |
| 93 | BAL | 0 | 37.60 | 0 | 0 | 0 | 0 | Bio-Rad | 0 | 0 | 0 |
| 94 | SP | 0 | 46.00 | 0 | 0 | 0 | 0 | Bio-Rad | 0 | 0 | 0 |
| 95 | BAL | 0 | 46.00 | 0 | 0 | 0 | 0 | Bio-Rad | 0 | 0 | 0 |
| 96 | BAL | 0 | 38.88 | 0 | 0 | 0 | 0 | Bio-Rad | 0 | 0 | 0 |
| 97 | BAL | 0 | 46.00 | 0 | 0 | 0 | 0 | Bio-Rad | 0 | 0 | 0 |
| 98 | SP | 0 | 46.00 | 0 | 0 | 0 | 0 | Bio-Rad | 0 | 0 | 0 |
| 99 | BAL | 0 | 46.00 | 0 | 0 | 0 | 0 | Bio-Rad | 0 | 0 | 0 |
| 100 | BAL | 0 | 46.00 | 0 | 0 | 0 | 0 | Bio-Rad | 0 | 0 | 0 |
| 101 | BAL | 0 | 46.00 | 0 | 0 | 0 | 0 | Bio-Rad | 0 | 0 | 0 |
| 102 | BAL | 0 | 46.00 | 0 | 0 | 0 | 0 | Bio-Rad | 0 | 0 | 0 |
| 103 | BAL | 0 | 46.00 | 0 | 0 | 0 | 0 | Bio-Rad | 0 | 0 | 0 |
| 104 | BAL | 0 | 46.00 | 0 | 0 | 0 | 0 | Bio-Rad | 0 | 0 | 0 |
| 105 | BAL | 43625 | 27.34 | 0 | 0 | 0 | 0 | Bio-Rad | 0 | 0 | 0 |
| 106 | SP | 0 | 46.00 | 0 | 0 | 0 | 0 | Bio-Rad | 0 | 0 | 0 |
| 107 | BAL | 0 | 46.00 | 0 | 0 | 0 | 0 | Bio-Rad | 0 | 0 | 0 |
| 108 | SP | 0 | 46.00 | 0 | 0 | 0 | 0 | Bio-Rad | 0 | 0 | 0 |
| 109 | BAL | 0 | 46.00 | 0 | 0 | 0 | 0 | Bio-Rad | 0 | 0 | 0 |
| 110 | SP | 0 | 46.00 | 0 | 0 | 0 | 0 | Bio-Rad | 0 | 0 | 0 |
| 111 | BAL | 0 | 46.00 | 0 | 0 | 0 | 0 | Bio-Rad | 0 | 0 | 0 |
| 112 | BAL | 262303 | 24.52 | 1 | 0 | 1 | 1 | Merifluor | 1 | 1 | 1 |
| 113 | SP | 0 | 46.00 | 1 | 0 | 0 | 1 | Merifluor | 0 | 0 | 0 |
| 114 | BAL | 817 | 33.58 | 0 | 0 | 0 | 0 | Merifluor | 0 | 0 | 0 |
| 115 | SP | 0 | 46.00 | 0 | 0 | 0 | 0 | Merifluor | 0 | 0 | 0 |
| 116 | BAL | 0 | 46.00 | 1 | 0 | 0 | 1 | Merifluor | 0 | 0 | 0 |
| 117 | BAL | 0 | 46.00 | 0 | 0 | 0 | 0 | Merifluor | 0 | 0 | 0 |
| 118 | BAL | 2184 | 32.04 | 0 | 0 | 0 | 0 | Merifluor | 0 | 0 | 0 |
| 119 | SP | 1900 | 32.26 | 0 | 0 | 0 | 0 | Bio-Rad | 0 | 0 | 0 |
| 120 | BAL | 0 | 46.00 | 0 | 0 | 0 | 0 | Merifluor | 0 | 0 | 0 |
| 121 | BAL | 0 | 46.00 | 0 | 0 | 0 | 0 | Bio-Rad | 0 | 0 | 0 |
| 122 | SP | 13178 | 29.22 | 0 | 0 | 0 | 0 | Bio-Rad | 0 | 0 | 0 |
| 123 | BAL | 0 | 46.00 | 0 | 0 | 0 | 0 | Bio-Rad | 0 | 0 | 0 |
| 124 | SP | 46168 | 28.34 | 0 | 0 | 0 | 0 | Bio-Rad | 0 | 0 | 0 |
| 125 | SP | 0 | 46.00 | 0 | 0 | 0 | 0 | Bio-Rad | 0 | 0 | 0 |
| 126 | SP | 0 | 46.00 | 0 | 0 | 0 | 0 | Bio-Rad | 0 | 0 | 0 |
| 127 | BAL | 0 | 46.00 | 0 | 0 | 0 | 0 | Bio-Rad | 0 | 0 | 0 |
| 128 | BAL | 0 | 46.00 | 0 | 0 | 0 | 0 | Bio-Rad | 0 | 0 | 0 |
| 129 | SP | 0 | 46.00 | 0 | 0 | 0 | 0 | Bio-Rad | 0 | 0 | 0 |
| 130 | BAL | 0 | 46.00 | 0 | 0 | 0 | 0 | Bio-Rad | 0 | 0 | 0 |
| 131 | SP | 0 | 46.00 | 0 | 0 | 0 | 0 | Bio-Rad | 0 | 0 | 0 |
| 132 | SP | 0 | 46.00 | 0 | 0 | 0 | 0 | Bio-Rad | 0 | 0 | 0 |
| 133 | BAL | 0 | 46.00 | 0 | 0 | 0 | 0 | Bio-Rad | 0 | 0 | 0 |
| 134 | BAL | 0 | 46.00 | 0 | 0 | 0 | 0 | Bio-Rad | 0 | 0 | 0 |
| 135 | BAL | 46964 | 33.55 | 0 | 0 | 0 | 0 | Bio-Rad | 0 | 0 | 0 |
| 136 | SP | 2160 | 41.31 | 0 | 0 | 0 | 0 | Bio-Rad | 0 | 0 | 0 |
| 137 | BAL | 441569 | 27.90 | 0 | 0 | 0 | 0 | Merifluor | 1 | 1 | 1 |
| 138 | BAL | 318229 | 28.73 | 0 | 0 | 0 | 0 | Merifluor | 0 | 1 | 1 |
| 139 | SP | 0 | 46.00 | 0 | 0 | 0 | 0 | Merifluor | 0 | 0 | 0 |
| 140 | BAL | 2458 | 40.98 | 0 | 0 | 0 | 0 | Merifluor | 0 | 0 | 0 |
| 141 | BAL | 0 | 46.00 | 0 | 0 | 0 | 0 | Merifluor | 0 | 0 | 0 |
| 142 | BAL | 0 | 46.00 | 0 | 0 | 0 | 0 | Merifluor | 0 | 0 | 0 |
| 143 | SP | 0 | 46.00 | 0 | 0 | 0 | 0 | Merifluor | 0 | 0 | 0 |
| 144 | BAL | 0 | 46.00 | 0 | 0 | 0 | 0 | Merifluor | 0 | 0 | 0 |
| 145 | BAL | 4252 | 39.60 | 0 | 0 | 0 | 0 | Merifluor | 0 | 0 | 0 |
| 146 | SP | 0 | 46.00 | 0 | 0 | 0 | 0 | Merifluor | 0 | 0 | 0 |
| 147 | SP | 0 | 46.00 | 0 | 0 | 0 | 0 | Merifluor | 0 | 0 | 0 |
| 148 | BAL | 0 | 46.00 | 0 | 0 | 0 | 0 | Merifluor | 0 | 0 | 0 |
| 149 | BAL | 100053 | 31.64 | 0 | 0 | 0 | 0 | Merifluor | 1 | 1 | 1 |
| 150 | BAL | 0 | 46.00 | 0 | 0 | 0 | 0 | Merifluor | 0 | 0 | 0 |
| 151 | BAL | 0 | 46.00 | 0 | 0 | 0 | 0 | Merifluor | 0 | 0 | 0 |
| 152 | BAL | 0 | 46.00 | 0 | 0 | 0 | 0 | Merifluor | 0 | 0 | 0 |
| 153 | SP | 8121545 | 22.31 | 0 | 0 | 0 | 0 | Merifluor | 0 | 1 | 1 |
| 154 | BAL | 0 | 46.00 | 0 | 0 | 0 | 0 | Merifluor | 0 | 0 | 0 |
| 155 | BAL | 1328500 | 25.13 | 1 | 1 | 1 | 1 | Merifluor | 1 | 1 | 1 |
| 156 | BAL | 46976 | 33.55 | 0 | 0 | 0 | 0 | Merifluor | 0 | 0 | 0 |
| 157 | BAL | 2305465 | 23.74 | 1 | 0 | 0 | 1 | Merifluor | 1 | 1 | 1 |
| 158 | BAL | 414727 | 28.06 | 0 | 0 | 0 | 0 | Merifluor | 0 | 0 | 0 |
| 159 | BAL | 2059340 | 24.02 | 1 | 0 | 1 | 1 | Merifluor | 1 | 1 | 1 |
| 160 | BAL | 0 | 46.00 | 0 | 0 | 0 | 0 | Merifluor | 0 | 0 | 0 |
| 161 | BAL | 0 | 46.00 | 0 | 0 | 0 | 0 | Merifluor | 0 | 0 | 0 |
| 162 | BAL | 14767 | 29.20 | 0 | 0 | 0 | 0 | Merifluor | 0 | 0 | 0 |
| 163 | BAL | 0 | 46.00 | 0 | 0 | 0 | 0 | Merifluor | 0 | 0 | 0 |
| 164 | BAL | 0 | 46.00 | 0 | 0 | 0 | 0 | Merifluor | 0 | 0 | 0 |
| 165 | SP | 0 | 46.00 | 1 | 0 | 0 | 1 | Merifluor | 1 | 1 | 0 |
| 166 | BAL | 0 | 46.00 | 0 | 0 | 0 | 0 | Merifluor | 0 | 0 | 0 |
| 167 | SP | 0 | 46.00 | 0 | 0 | 0 | 0 | Merifluor | 0 | 0 | 0 |
| 168 | BAL | 15386 | 36.36 | 0 | 0 | 0 | 0 | Merifluor | 0 | 0 | 0 |
| 169 | SP | 0 | 46.00 | 1 | 0 | 0 | 1 | Merifluor | 1 | 1 | 0 |
| 170 | BAL | 81125 | 32.17 | 0 | 0 | 0 | 0 | Merifluor | 0 | 0 | 0 |
| 171 | BAL | 0 | 46.00 | 0 | 0 | 0 | 0 | Merifluor | 0 | 0 | 0 |
| 172 | SP | 7996525 | 20.61 | 1 | 0 | 0 | 1 | Merifluor | 1 | 1 | 1 |
| 173 | BAL | 4117614 | 22.28 | 1 | 1 | 1 | 1 | Merifluor | 1 | 1 | 1 |
| 174 | BAL | 0 | 46.00 | 0 | 0 | 0 | 0 | Merifluor | 0 | 0 | 0 |
| 175 | BAL | 0 | 46.00 | 0 | 0 | 0 | 0 | Merifluor | 0 | 0 | 0 |
| 176 | BAL | 0 | 46.00 | 0 | 0 | 0 | 0 | Merifluor | 0 | 0 | 0 |
| 177 | BAL | 31622777 | 16.18 | 1 | 1 | 1 | 1 | Merifluor | 1 | 1 | 1 |
| 178 | BAL | 0 | 46.00 | 0 | 0 | 0 | 0 | Merifluor | 0 | 0 | 0 |
| 179 | SP | 0 | 46.00 | 0 | 0 | 0 | 0 | Merifluor | 0 | 0 | 0 |
| 180 | SP | 0 | 46.00 | 0 | 0 | 0 | 0 | Merifluor | 0 | 0 | 0 |
| 181 | BAL | 72919 | 32.44 | 0 | 0 | 0 | 0 | Merifluor | 0 | 0 | 0 |
| 182 | BAL | 0 | 46.00 | 0 | 0 | 0 | 0 | Merifluor | 0 | 0 | 0 |
| 183 | BAL | 0 | 46.00 | 0 | 0 | 0 | 0 | Merifluor | 0 | 0 | 0 |
| 184 | BAL | 350112 | 28.49 | 1 | 1 | 0 | 1 | Merifluor | 1 | 1 | 1 |
| 185 | BAL | 0 | 46.00 | 0 | 0 | 0 | 0 | Merifluor | 0 | 0 | 0 |
| 186 | BAL | 76283 | 32.33 | 0 | 0 | 0 | 0 | Merifluor | 0 | 0 | 0 |
| 187 | BAL | 154 | 37.28 | 1 | 0 | 0 | 1 | Merifluor | 1 | 1 | 1 |
| 188 | BAL | 0 | 46.00 | 0 | 0 | 0 | 0 | Merifluor | 0 | 0 | 0 |
| 189 | BAL | 0 | 46.00 | 0 | 0 | 0 | 0 | Merifluor | 0 | 0 | 0 |
| 190 | BAL | 0 | 39.30 | 0 | 0 | 0 | 0 | Merifluor | 0 | 0 | 0 |
| 191 | BAL | 0 | 46.00 | 0 | 0 | 0 | 0 | Merifluor | 0 | 0 | 0 |
| 192 | SP | 0 | 46.00 | 0 | 0 | 0 | 0 | Merifluor | 0 | 0 | 0 |
| 193 | BAL | 0 | 46.00 | 1 | 0 | 0 | 1 | Merifluor | 1 | 1 | 0 |
| 194 | BAL | 0 | 46.00 | 0 | 0 | 0 | 0 | Merifluor | 0 | 0 | 0 |
| 195 | BAL | 0 | 46.00 | 0 | 0 | 0 | 0 | Merifluor | 0 | 0 | 0 |
| 196 | SP | 0 | 40.49 | 0 | 0 | 0 | 0 | Merifluor | 0 | 0 | 0 |
| 197 | BAL | 0 | 46.00 | 0 | 0 | 0 | 0 | Merifluor | 0 | 0 | 0 |
| 198 | BAL | 0 | 46.00 | 0 | 0 | 0 | 0 | Merifluor | 0 | 0 | 0 |
| 199 | BAL | 19740 | 28.75 | 0 | 0 | 0 | 0 | Merifluor | 0 | 0 | 0 |
| 200 | BAL | 5644021 | 20.06 | 0 | 0 | 0 | 0 | Merifluor | 0 | 1 | 1 |
| 201 | BAL | 68523 | 27.09 | 1 | 0 | 0 | 1 | Merifluor | 1 | 1 | 1 |
| 202 | BAL | 102 | 36.93 | 0 | 0 | 0 | 0 | Merifluor | 0 | 0 | 0 |
| 203 | BAL | 6345 | 30.36 | 1 | 0 | 1 | 0 | Bio-Rad | 1 | 1 | 1 |
| 204 | BAL | 0 | 46.00 | 0 | 0 | 0 | 0 | Bio-Rad | 0 | 0 | 0 |
| 205 | BAL | 0 | 46.00 | 0 | 0 | 0 | 0 | Bio-Rad | 0 | 0 | 0 |
| 206 | SP | 0 | 46.00 | 0 | 0 | 0 | 0 | Bio-Rad | 0 | 0 | 0 |
| 207 | SP | 0 | 46.00 | 0 | 0 | 0 | 0 | Bio-Rad | 0 | 0 | 0 |
| 208 | BAL | 0 | 46.00 | 0 | 0 | 0 | 0 | Bio-Rad | 0 | 0 | 0 |
| 209 | SP | 0 | 46.00 | 0 | 0 | 0 | 0 | Bio-Rad | 0 | 0 | 0 |
| 210 | SP | 0 | 46.00 | 0 | 0 | 0 | 0 | Bio-Rad | 0 | 0 | 0 |
| 211 | SP | 301 | 35.15 | 0 | 0 | 0 | 0 | Bio-Rad | 0 | 0 | 0 |
| 212 | BAL | 0 | 46.00 | 0 | 0 | 0 | 0 | Bio-Rad | 0 | 0 | 0 |
| 213 | SP | 0 | 46.00 | 0 | 0 | 0 | 0 | Bio-Rad | 0 | 0 | 0 |
| 214 | BAL | 0 | 46.00 | 0 | 0 | 0 | 0 | Bio-Rad | 0 | 0 | 0 |
| 215 | SP | 154 | 36.20 | 0 | 0 | 0 | 0 | Bio-Rad | 0 | 0 | 0 |
| 216 | BAL | 0 | 46.00 | 0 | 0 | 0 | 0 | Bio-Rad | 0 | 0 | 0 |
| 217 | BAL | 0 | 46.00 | 0 | 0 | 0 | 0 | Bio-Rad | 0 | 0 | 0 |
| 218 | BAL | 0 | 46.00 | 0 | 0 | 0 | 0 | Bio-Rad | 0 | 0 | 0 |
| 219 | BAL | 0 | 46.00 | 0 | 0 | 0 | 0 | Bio-Rad | 0 | 0 | 0 |
| 220 | SP | 6121 | 30.42 | 0 | 0 | 0 | 0 | Bio-Rad | 0 | 0 | 0 |
| 221 | BAL | 0 | 46.00 | 0 | 0 | 0 | 0 | Bio-Rad | 0 | 0 | 0 |
| 222 | BAL | 0 | 46.00 | 0 | 0 | 0 | 0 | Bio-Rad | 0 | 0 | 0 |

Abbreviations: NR, sample number; MAT, material; Gie, Giemsa; Tol, Toluidine blue; IFA, IF assay; RXM, result after reexamination; CR, result after clinical reevaluation; NFN, no false negatives

Table S2: Detailed clinical information of patients with IF+/PCR− and IF−/PCR+ results.

a) IF+/PCR-

| Re | ST | IFA | Host factor | Prophx | PJP therapy | Clinical course; treatment response | Additional Info |
| --- | --- | --- | --- | --- | --- | --- | --- |
| Possible or probable PJP |
| NA | SP | Mf | GC inh. if required due to COPD GOLD 3; MDS (unlikely host factor per oncologists) | - | th:TMP-SMX | Suitable clinical presentation and radiology; adequate | Oral candidiasis (Fluconazole treatment), HSV1-reactivation |
| Unclear |
| neg | BAL | BR | SCID (Severe Combined Immunodeficiency) | Bactrim | th:TMP-SMX | Suitable clinical presentation; unclear | 2d post-SC after revisiting the same sample: only cyst not found anymore |
| neg | BAL | Mf | s/p heart-TPL: MMF, Tac, Ever | Nopil | th:TMP-SMX | Suitable clinical presentation; adequate | Poor treatment response to Co-Amoxicillin |
| NA | SP | Mf | Pred p.o. 15mg/d due to lymphocytic pneumonia | Bactrim | th:TMP-SMX 1d until death | Suitable clinical presentation and radiology; unclear (death: 2d post-SC) | St.n. Pneumocystis jirovecii pneumonia, ca. 90d pre-SC (PCR +) |
| NA | BAL | Mf | GC i.v. 60mg/d from 3d-preSC – death; severe underlying multisystem disease | - | th:TMP-SMX 10d until death | Decompensated cardiac insufficiency + congestive and chronic hepatopathy;  unclear (death: 10d post-SC, septic and hemorrhagic shock ) | E.coli (4/4): BC (16d pre-SC), C. dubliniensis: UC (11d pre-SC) +  BAL (present sample), Yeast fungus: UC (5d post-SC) |
| Alternative diagnosis |
| neg | SP | Mf | Pred p.o. (≤10mg/d) if required due to COPD; SCLC (limited disease, no treatment) | - | th:TMP-SMX 7d until death | Suitable clinical presentation, radiologically unclear; poor (death 7d post-SC) | S. aureus found in the sputum sample, additional to PJ |

All sample collections took place in 2023. 

Abbreviations: Re, result after Reexam; ST, sample type; SP, sputum; IFA, IF assay; BR, Bio-Rad; Mf, Merifluor; Prophx, PJP prophylaxis;  d, day(s); SC, day of sample collection; pre-SC, before sample collection; post-SC, after sample collection; s/p, status post; inh., inhalative; p.o., peroral; i.v., intravenous; th, therapeutic; GC, Glucocorticoids; Pred, Prednisone; MMF, Mycophenolat mofetil; Tac, Tacrolimus; Ever, Everolimus; BC, blood culture; UC, urine culture; SCLC, small cell lung cancer

b) IF-/PCR+ (>100'000 cp/ml or Ct value <29; data not available for three patients)

| Re | ST | IFA | Host factor | Prophx | PJP therapy | Clinical + Outcome | Additional Info |
| --- | --- | --- | --- | --- | --- | --- | --- |
| Definite PJ diagnosis |
| neg | BAL | Mf | s/p kidney-TPL: Tac, MMF, Pred, induction anti-T-Lyc-globulin-Ig | - | th:TMP-SMX 6d: from 13d–7d pre-SC;  Clinda + PQ 7d pre-SC–10d post-SC; afterwards Atovaq (pr) | Definite PJP diagnosis (13d pre-SC), 16d pre-SC suitable radiology and improvement seen on day of sample collection | Positive with IFA + TOL + PCR; Aspergillus: BAL (13d pre-SC), P. aeruginosa: TS (16d pre-SC) |
| NA | SP | Mf | s/p combined kidney- and liver-TPL: Tac, MMF, Pred 5mg, induction BXM | - | th:TMP-SMX 21d: SC–21d post-SC | Definite PJP diagnosis (2d post-SC), suitable radiology; 10d post-SC clinical improvement | Commentary: “questionable trophozoites found; re-sampling suggested”; 2d post-SC: Follow-up BAL sample IFA positive; Pred 40mg |
| neg | BAL | Mf | HIV1 C3 with opportunistic infections due to malcompliance; (HIV PCR = 405'000 cp/ml, CD4 = 183 cells/μl) | - | th:TMP-SMX 21d: 1d pre-SC–20d post-SC | Definite PJP diagnosis (1d pre-SC), suitable radiology; later clinical improvement | Fluconazole (6d–9d post-SC for oral candidiasis), Pred (1d pre-SC–20d post-SC); External PCR (2*106 cp/ml) 3d post-SC, follow-up IF with new staining still negative |
| Possible or probable PJP |
| neg | SP | BR | Large T cell lymphoma: MPred 80mg (1d pre-SC) | - | th:TMP-SMX 5d: SC–5d post-SC; afterwards prophylactic | Lower abdominal pain at admission; development of type 1 respiratory failure, 5d post-SC clinical improvement, 7d post-SC develops fever again; 19d post-SC discharge | 1d pre-SC: MPred 80mg d1 + 160mg d2 From SC: MPred 100mg, ETO, CDV, G-CSF;  HSV1-Reaktivierung (7d post-SC), E. faecium: UC (7d post-SC) |
| pos | BAL | Mf | s/p stem cell-TPL: acute fibrinous and organizing pneumonia most likely due to GvHD II | Bactrim, until 90d pre-SC | pr:TMP-SMX 7d post-SC–while high-dose GC therapy | Suitable clinical presentation and radiology, LDH↑; 8d post-SC clinical improvement | MPred i.v.125mg (4d–7d post-SC), Pred 120mg (7d post-SC–XX); Adenovirus: PCR + |
| neg | BAL | Mf | s/p heart-TPL: MMF, Tac, Ever, Pred 2.5mg, induction BXM; dialysis-dependent CKD | - | th:TMP-SMX 6d: 1d pre-SC–5d post-SC | Suitable clinical presentation and radiology; 11d post-SC clinical improvement | Ceftriaxon 6d–1d pre-SC |
| Alternative diagnosis |
| NA | SP | BR | GCA: Pred p.o. 60mg/d (46d pre-SC) - 20mg (ca. 30d post-SC); Gout: Pred p.o. 50mg if required; dialysis-dependent CKD | Bactrim | pr:TMP-SMX 1d pre-SC–21d post-SC | Suspected COVID-19 pneumonia with possible bacterial superinfection; 10d post-SC clinical improvement | Cefepim 1g (1d pre-SC), Nirmatrelvir/Ritonavir (1d pre-SC–3d post-SC), Metronidazol (SC–3d post-SC) |
| neg | BAL | Mf | s/p kidney-TPL: CyA, MMF, dialysis-dependent CKD | - | pr:TMP-SMX 9d post-SC–while high-dose GC therapy | Cryptogenic organizing pneumonia (ED 7d post-SC); 9d post-SC clinical improvement | Co-Amoxicillin (1d pre-SC – 13d post-SC), Pred 30mg + Amphotericin B (11d post-SC–XX) |
| Unclear |
| NA | SP | BR | Autoimmune hepatitis: MPred i.v. 100mg (13d pre-SC–7d pre-SC), Pred p.o. 75mg (6d pre-SC–death), Aza (6d pre-SC–death) | - | - | Unclear (acute Hepatitis, respiratory insufficiency); unclear (death: 4d post-SC, septic shock) | S. aureus: BC (4/4) (2d post-SC), UC+BC (1/4) (SC); A. fumigatus: SP (1d post-SC); Vancomycin i.v. (3d pre-SC),  Piperacillin/Tazobactam (3d pre-SC – death) |
| NA | SP | BR | s/p heart-TPL: Tac, MMF, Pred 5mg | - | th:TMP-SMX 2d: 5d–7d post-SC | Decreasing kidney function, Suitable clinical presentation and radiology, LDH↑; 5d post-SC pulmonary deterioration; 11d post-SC clinical improvement | 3 additional samples (IFA-/PCR+): 2x SP (ca. 1d post-SC), BAL (7d post-SC); Meropenem (6d–10d post-SC), Levofloxacin (1d–3d post-SC + 6d–14d post-SC), Co-Amoxicillin (9d–14d post-SC) |

All sample collections took place in 2023 besides one marked with (*). All dates mentioned in one bracket refer to the same year as the sample collection.

Abbreviations: Re, result after Reexam; ST, sample type; IFA, IF assay; Prophx, PJP prophylaxis; d, day(s); SC, day of sample collection; pre-SC, before sample collection; post-SC, after sample collection; s/p, status post; inh., inhalative; p.o., peroral; i.v., intravenous; th, therapeutic; pr, prophylactic; PQ, Primaquine; Clinda, Clindamycin; GC, Glucocorticoids; Pred, Prednisone; MPred, Methylprednisone; Aza, Azathioprine; CyA, Cyclosporin A; Tac, Tacrolimus; MMF, Mycophenolat mofetil; BXM, Basiliximab; ETO, Etoposid; CDV, Cyclophosphamid + Doxorubicin + Vincristin; Ever, Everolimus; d., death; BC, blood culture; UC, urine culture; TS, tracheal secretion; CDK, chronic kidney disease, GCA, giant cell arteritis; XX, unknown date
